# Will Perceived Risk of COVID-19 Move Exhibition Visitors from On-Site to Virtual? Focusing on Exhibition Quarantine Service Quality and Switching Intention

**DOI:** 10.3390/ijerph19116388

**Published:** 2022-05-24

**Authors:** Mi-Hwa Jang, Eui-Yul Choi

**Affiliations:** 1 Department of Industry-University Cooperation, Keimyung University, Daegu 42601, Korea; pw332@daum.net; 2 Department of Marine Sport Sciences, Korea Maritime & Ocean University, Busan 49112, Korea

**Keywords:** COVID-19, perceived risk, new exhibition environment, COVID-19 quarantine service quality, switching intention

## Abstract

COVID-19 has shifted people’s activities from the real world to the virtual world in many fields, such as conferences, shopping, education, and more. In the field of MICE, however, exhibitions have been held steadily since the second half of 2020 in the form of on-site exhibitions. The exhibition organizers and related authorities have tried to attract exhibitors and visitors to the exhibition hall by providing exhibition quarantine services. Moreover, despite various perceived risks during the COVID-19 period, exhibition visitors continue to visit the exhibition. This study, therefore, paid attention to the psychological factors of visitors who consistently visit on-site exhibitions even during the pandemic. In addition to the perceived risks, this study tried to examine the quality of exhibition quarantine services and switching intention of visitors, and to analyze the relationships between them. A survey of 167 people who visited the camping exhibition and well-food exhibition held in June 2021 found that they would not visit the exhibition due to the functional and financial risk of the exhibition rather than the risk of the virus. On the other hand, it was found that visitors who felt the social risk of COVID-19 valued the quality of exhibition quarantine service. Furthermore, the study found that the quarantine service quality lowered switching intention. Therefore, the study suggests that exhibition organizers should think about ways to strengthen the most essential characteristics of on-site exhibitions along with appropriate quarantine measures to induce steady visits even during the pandemic.

## 1. Introduction

In January 2022, CES, the world’s largest consumer electronics exhibition, held an on-site exhibition on a reduced scale, and the largest display exhibition in Europe, ISE, was postponed to March 2023. As such, the COVID-19 virus affected related industrial entities such as MICE (Meeting, Incentives, Convention, and Exhibition) and tourism by restricting movement to other regions, maintaining an appropriate distance between people, and restricting group activities, and further, is severely damaging the national economy and the global economy [1,2]. In Korea, most exhibitions were minimized or postponed for about 4 months right after the declaration of a pandemic, where the new virus was not well understood and there were no quarantine guidelines for exhibitions [3]. In particular, the psychological burden of visit in the MICE industry, which has created various values through direct meeting and exchange activities, and the prohibition of exhibitions and gatherings made it unclear whether MICE was held. However, in recent years in Korea, exhibitions as economic activities have been held steadily since the second half of 2020 due to the efforts of the exhibition industry’s key players and the set of appropriate quarantine rules for exhibitions, and certain exhibitions have attracted enough people to overshadow the pandemic. In the field of MICE, meetings are actively being held using virtual spaces. For exhibitions, however, the analysis continues that virtual spaces have not replaced on-site exhibition, and this will continue in the future [4,5]. This can be interpreted as it being difficult for virtual spaces to realize the intrinsic value provided by on-site exhibitions [2,4].

In general, when people perceive a risk before taking a certain action, especially tourism, they are more likely to postpone or cancel the action [6,7]. During previous epidemics such as SARS, H1N1 flu, and MERS, it was found that people’s perception of the risk of infectious diseases influenced their behavior [8,9,10]. Even in the recent COVID-19 era, it has been found that the perceived risk in several fields influences the formation of intentions to lower purchase intentions or switch to alternatives [11,12,13,14,15,16,17,18,19]. However, as mentioned above, on-site exhibitions are still preferred as a channel for networking and accumulation of experiences, and the research results show that more than 80% of exhibitors and visitors will participate in on-site exhibitions, most exhibitions in Korea are being held from the second half of 2020 [5]. Therefore, we need to pay attention to the psychological factors of visitors to exhibitions even in the pandemic where there is a risk of infection with the virus and there is no choice but to meet an unspecified number of people.

Therefore, this study was conducted on perceived risk, COVID-19 quarantine service quality, and switching intention of visitors to the camping exhibition and well-food exhibition held at EXCO in Daegu during the second step of the Korean coronavirus quarantine from 25–27 June 2021. The study had the following detailed objectives: (1) to analyze the effect of perceived risk on COVID-19 quarantine service quality; (2) to analyze the effect of perceived risk on switching intention; and (3) to analyze the effect of COVID-19 quarantine service quality on switching intention regarding COVID-19. As such, this study aimed to understand the psychological factors of exhibition visitors, and further to suggest academic and practical implications for deriving incentives for more exhibitors and visitors to participate in exhibitions classified as essential economic activities even in the global pandemic.

## 2. Literature Review and Research Question Development

### 2.1. Perceived Risk

In general, risk is divided into absolute risk, actual risk, and perceived risk [9,20]. Among them, perceived risk refers to the perception of uncertainty and negative consequences prior to purchase when consumers want to purchase a product or service [9,21,22]. Unlike actual risk, the perceived risk is a subjective risk that can be felt differently by each person [23]. In other words, when consumers plan to purchase a product, when tourists plan to travel, or when visitors plan to visit an exhibition, it is the potential and subjective risk that people perceive unfamiliar situations before taking action. Perceived risk has been measured in various ways depending on researchers and study subjects. Sönmez and Graefe [24] measured the perceived risks as instability regarding financial, functional, physical, psychological, social, and political factors. Mäser and Weiermair [25] presented uncertainties due to disease, crime, natural disaster, accident, sanitation, transportation risk, cultural/language barrier, and regulation and law of travel destination. In addition, environment, time loss, terrorist risk, satisfaction, and exchange rate were added and measured as those perceived [26,27,28,29].

Several previous studies found that customers had a high tendency to postpone or cancel subsequent actions when they perceived a certain risk in advance [6,7]. In particular, many previous studies regarding infectious diseases such as SARS, H1N1 flu, and MERS found that the risk perception of the diseases affected customers’ behavior [8,9,10]. Even in the recent COVID-19 era, it has been confirmed that the perceived risk of coronavirus in several fields influences the formation of intentions to reduce purchase behaviors or switch to alternatives. Habib and Hamadneh [1], Pham, Do Thi and Ha Le [18], and Martinell, De Canio, and Nardin [14] found that the fear of COVID-19 is causing a large shift in consumer purchasing activity from offline to online. A study by Hakim, Zanetta, and Cunha [12] found that the perceived risk of COVID-19 influenced the intention to revisit restaurants. In addition, in a post-COVID-19 crisis travel behavior study, Matiza [15] found that perceived risk associated with the pandemic affected the post-crisis behavior of tourists. Ozbilen, Slagle, and Akar [16] and Jittrapirom and Tanaksaranond [13] reported that a perceived risk of infection by COVID-19 promoted customers to move to an on-line platform such as for shopping and teleconferencing. A study by Sánchez-Cañizares, Cabeza-Ramírez, Muñoz-Fernández, and Fuentes-García [19] found that the perceived risk of COVID-19 negatively affected travel intentions. Moreover, Palau-Saumell, Matute, Derqui, and Meyer [17] found that the perceived risk of COVID-19 influenced customers’ willingness to visit theme parks.

As found in previous studies, the conference field among the MICE industry is also being largely replaced by online conferences and hybrid forms that avoid viruses and participate in one’s hideout. On the other hand, on-site exhibition, which has the core value of direct product experience, is still preferred in the midst of the pandemic, and it is predicted that this phenomenon of exhibition will continue in the future [5]. Here, we need to pay attention to the psychological variables of visitors to the exhibition even though they are aware of the risk, unlike previous studies in which the perceived risk of COVID-19 changes customer behavior. In addition to the perceived risk associated with COVID-19 of the exhibition visitors, therefore, it is necessary to take a concrete approach to the awareness of the risk directly related to the exhibition and the environment of the changed exhibition reflecting the efforts to resolve the above risks. That may reduce their switching intention of visitors that they will not visit the exhibition again.

### 2.2. Exhibition Environment Change Due to COVID-19, ‘COVID-19 Quarantine Service Quality’

People are living a life of coexistence with the virus in the COVID-19 era by maintaining social distance and following quarantine rules. In the MICE industry, various countries and related organizations around the world are making efforts to normalize on-site MICE events by producing and distributing guidelines and crisis management manuals to operate on-site MICE according to the COVID-19 situation. The Global Association of the Exhibition Industry (UFI), the International Convention Center Association (AIPC), and the International Convention Association (ICCA), which are representative international organizations in the MICE industry, jointly prepared as the quarantine guideline ‘The Addressing COVID-19 Requirements for Re-Opening Business Event’ [30,31]. In Korea, in order to hold on-site MICE as safely as possible from viruses, governments and organizations provide quarantine guidelines such as the Ministry of Health and Welfare’s guidelines for group event quarantine management, the Seoul Metropolitan Government’s MICE event infectious disease management guidelines, and the Korea Exhibition Industry Promotion Association’s exhibition quarantine management acts [2].

Under these guidelines, on-site exhibitions provide visitors with a new exhibition service quality, a previously unfamiliar environment, such as restrictions on entry for distancing, temperature checks, and disinfection procedures. The service quality of the exhibition is a subjective evaluation of the visitors’ perception of the overall service provided by the exhibition organizers [32], which can be a key factor leading to the visitors’ future visit. The theory suggested by Zeithaml, Berry, and Parasuraman [33] that service quality affects customers’ favorable or unfavorable behavior has been found in exhibition research [34,35,36,37,38]. In particular, a study by Hakim, Zanetta, and Cunha [12] found that the perceived risk of COVID-19, disease denial, and health surveillance trust in restaurants influence the intention to revisit restaurants, which provides great implications for exhibition organizers. In the COVID-19 era, therefore, ‘COVID-19 quarantine service quality’ of exhibition provided to reduce the risk of virus infection can be an important factor in choosing whether to visit an exhibition or not.

### 2.3. Switching Intention

Switching intention is a factor that induces switching behavior and means the will to change from a specific product or service to another new one [39]. This is an opposite concept to repurchase intention or continuous purchase intention and is the intention of service users to deviate from one service to another [40,41]. It can be predicted that the stronger the switching intention, the higher the switching behavior [42,43]. Since switching intention is an important predictor that can cause economic damage to service providers due to customer loss, various prior studies have been conducted on the relationship with perceived risk, service quality, and ethical issues, as antecedent variables [17,41,43,44,45,46,47,48]. In particular, Oh et al. [28] emphasized that analyses of factors causing switching behavior should be preceded since MICE participants’ switching intention can give fatal results in reduced profits, reduced competitiveness, and so on. Recently, Martinell, De Canio, and Nardin [14] found that fear of COVID-19 further reinforces the intention to switch from offline shopping to online shopping. As such, it can be predicted that perceived risks and service quality associated with recent exhibitions, such as COVID-19, may reinforce the unfavorable will of visitors to plan to visit exhibitions.

**Figure 1:**
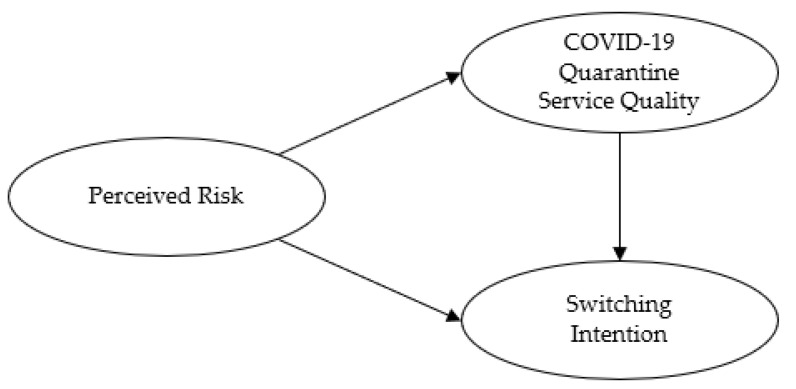
Theoretical framework on the relationship between perceived risk, COVID-19 quarantine service quality, and switching intention of on-site exhibition visitors.

Therefore, this study aimed to derive factors for expanding visitors to on-site exhibitions by studying the relationship between risk factors perceived by visitors to on-site exhibitions, COVID-19 quarantine service quality, and switching intention for visiting exhibitions. In order to accomplish the study purposes, we set out the following three research questions and theoretical framework, which we based on the previous studies that have been cited above (see Figure 1):

Research Question 1: Does perceived risk affect COVID-19 quarantine service quality?

Research Question 2: Does perceived risk affect switching intention?

Research Question 3: Does COVID-19 quarantine service quality affect switching intention?

## 3. Materials and Methods

### 3.1. Subject of Survey

A survey for visitors of camping exhibition and well-food exhibition held in EXCO (Daegu Exhibition and Convention Center, Daegu, Korea), Daegu metropolitan city of South Korea, was conducted to analyze the relationship between perceived risk, COVID-19 quarantine service quality, and switching intention. The surveyors stayed at EXCO for three days from 25–27 June 2021, and distributed questionnaires to the participants who voluntarily expressed their intention to participate. They were informed of the purpose of the study and the importance of their participation and were guaranteed anonymity and confidentiality in completing the survey. A total of 202 questionnaires were distributed and 180 copies were collected, and 167 copies were used for analysis, excluding invalid questionnaires such as insincere responses. Of the 167 participants, 55 were men (32.9%) and 112 were women (67.1%). By age, 69 of the participants were in their 20s (41.3%), 45 were in their 30s (26.9%), 25 were in their 40s (15.0%), and 28 were over their 50s (16.8%). Looking at the number of visits, 88 participants visited 1–2 times (52.7%), 72 3–5 times (43.1%), and 7 visited 6 times or more (4.2%).

### 3.2. Measures

Perceived risk of the exhibition in the era of COVID-19 was derived from 4 factors and 19 items based on previous studies [24,25,26,27,28,29] (see Table 1). Functional risk is a functional defect of a product, defined as uncertainty about the basic quality of an exhibition, financial risk is a risk of money loss and time wasted, physical risk is a COVID-19 risk, and social risk is a perceived risk due to relationships with others. The items of the perceived risk were measured on a 5-point Likert scale, ranging from 1 (strongly disagree) to 5 (strongly agree).

COVID-19 quarantine service quality was measured by the ‘Quarantine and Safety Management Attributes’ developed and verified by Park and Hwang [2] based on the COVID-19 quarantine rules of the Korea Exhibition Industry Promotion Association, the world’s top 3 MICE organizations including UFI. The items of the COVID-19 quarantine service quality (see Table 2) were measured on a 5-point Likert scale, ranging from 1 (strongly disagree) to 5 (strongly agree).

In this study, switching intention can be defined as the will to make another choice instead of visiting the exhibition. The switching intention was modified with 1 factor and 6 items (Table 3) for the exhibition based on previous studies [17,41,43,44,45,46,47,48]. The items of the switching intention were measured on a 5-point Likert scale, ranging from 1 (strongly disagree) to 5 (strongly agree).

### 3.3. Data Processing

The data collected for this study were analyzed using SPSS 26.0 statistical program as follows. Exploratory factor analysis was conducted to derive the factors of perceived risk of exhibition in the era of COVID-19. Cronbach’s alpha coefficient was estimated to verify reliability of measuring tools for perceived risk, COVID-19 quarantine service quality, and switching intention. After this process, descriptive statistics for all the variables were computed. Finally, multiple regression analyses were performed to predict switching intention with perceived risk and COVID-19 quarantine service quality. All the statistical verifications were based on a significance level of 0.05.

## 4. Results

### 4.1. Reliability and Validity

Exploratory factor analysis on perceived risk (19 items) was performed using principal component analysis with Varimax rotation method. Two items that hindered unidimensionality or did not satisfy the factor loading of 0.5 or higher were dropped. The perceived risk consisted of four factors, namely, functional, financial, physical, and social risk. Each factor with the eigenvalue greater than 1 contained 3 to 5 items. Table 4 showed that these four factors explained 72.718% of total variance and a total of 17 items were converged. Cronbach’s α was located between 0.791 and 0.919, indicating that data were found to have high internal consistency [49]. Additionally, as a single factor, COVID-19 quarantine service quality and switching intention, data were found to have sufficient reliability according to the Cronbach’s α of 0.927 and 0.920, respectively.

### 4.2. Descriptive Statistics and Correlation Analysis

The exhibition visitors did not show a clear difference in perceived risk, but the highest mean value was shown by social risk (M = 2.936, SD = 0.895) and it was followed by physical risk (M = 2.889, SD = 0.817), financial risk (M = 2.874, SD = 0.736), and functional risk (M = 2.711, SD = 0.754). In addition, COVID-19 quarantine service quality (M = 3.596, SD = 0.475) was found to be stronger than switching intention (M = 2.145, SD = 0.743). Correlation analysis showed a negative relationship between physical risk and COVID-19 quarantine service quality and between COVID-19 quarantine service quality and switching intention. Meanwhile, in the correlation between all variables, the maximum coefficient value was 0.404, indicating that the discriminant validity between the variables was generally satisfied (see Table 5).

### 4.3. Effect of Perceived Risk on COVID-19 Quarantine Service Quality

Multiple regression analysis was conducted to explain the effect of perceived risk on COVID-19 quarantine service quality. As shown in the results presented in Table 6, among the factors in perceived risk, social risk positively predicted COVID-19 quarantine service quality, whereas the results for functional, financial, and physical risk were statistically nonsignificant. In regard to explanatory power, the model accounted for 8.2% of the variance in COVID-19 quarantine service quality. In addition, VIFs (Variance Inflation Factors) ranged from 1.131 to 2.270, respectively, which indicated that multicollinearity did not exist among the independent variables [50].

### 4.4. Effect of Perceived Risk on Switching Intention

Multiple regression analysis was also used to predict switching intention with perceived risk factors. As shown in the results presented in Table 7, functional risk and financial risk showed positive effects on switching intention. Physical risk and social risk did not statistically significantly influence switching intention. The variance in switching intention explained by the model was 15.2%.

### 4.5. Effect of COVID-19 Quarantine Service Quality on Switching Intention

Multiple regression analysis was conducted to explain the effect of COVID-19 quarantine service quality on switching intention. As shown in the results presented in Table 8, COVID-19 quarantine service quality negatively predicted switching intention. In regard to explanatory power, the model accounted for 9.9% of the variance in switching intention.

## 5. Conclusions

First of all, the biggest achievement of this study is to reconfirm the core value of the on-site exhibition. Now in the course of the pandemic, several researchers have explained that a lot of people’s activities are moving from offline to online [11,13,14,18]. In addition, in order to prepare a new industrial ecosystem in a virtual space, people are contemplating the development and industrial application of metaverse technologies such as VR and AR. On the other hand, UFI [5] explains in the ‘Global Recovery Insights Report’ that there is no evidence that the world’s exhibition industry is moving from the real world to the digital world even during the pandemic. Rather, UFI [5] found that on-site exhibitions are still preferred as a channel for networking and accumulation of experiences, and emphasized that more than 80% of exhibitors and visitors will participate in on-site exhibitions. This can also be found from the results of this study that fear of COVID-19 does not affect the intention not to visit exhibitions, but rather the functional and financial risk of exhibitions affect switching intention. Therefore, while it is important for exhibition organizers to technically understand virtual or hybrid exhibition, it is more important to prepare a way to improve functional and financial quality of on-site exhibition even in the time of coexistence with the virus.

Second, perceived social risk is the only factor that affects COVID-19 quarantine service quality. Until now, there have been few cases of second spread through exhibitions in Korea, but if it is classified as close contacts according to Korea’s quarantine rules, even asymptomatic people have the burden of self-quarantine, including coronavirus tests. Moreover, the fact that exhibition quarantine service quality is more important to those who are aware of the risk of harm to the people around them rather than the risk of their own infection has great implications for exhibition organizers. Therefore, exhibition organizers will need to remove risk factors for exhibition visitors by sharing cases that did not lead to the second spread of the virus through visiting exhibitions.

Third, this study derived specific items of quarantine management of exhibition organizers to properly respond to COVID-19, and confirmed its importance. This supports a study by Hakim, Zanetta, and Cunha [12] that the quality of quarantine service affects customers’ reuse intention. In particular, in this study, the result that exhibition environment changes due to COVID-19, ‘COVID-19 quarantine service quality’ has a negative (−) effect on switching intention is very important. Well-prepared exhibition quarantine acts play a key role in holding sustainable exhibitions by reducing the switching intention of visitors not to visit on-site exhibitions. Therefore, although the world is experiencing a time of confusion due to the unprecedented spread of the virus, it is necessary to consider the specificity of whether the object is the area of choice or the area of necessity such as exhibitions which are economic activities, not the uniform limit such as gathering restriction or inability to hold events in the future. As such, in the field of essential economic activities, it is necessary to prepare a plan for sustainable hosting through appropriate quarantine measures.

This study provides important implications for how the form of exhibitions will change during the course of the COVID-19 pandemic or in the era of post-COVID-19, but it also has some limitations. First, for generalization of study results, it is necessary to expand the scope of study subjects. Since the survey was conducted at the exhibition halls during the second step of social distancing in Korea, contact with visitors was not completely free, so the number of survey subjects is not large. It is necessary to additionally investigate visitors with changes in their behavior, who have been visiting the exhibition continuously but do not visit the exhibition due to COVID-19 fear. In addition, since this study was conducted with only two exhibitions, it would be necessary to expand to a variety of exhibition visitors considering the characteristics of visitors depending on the types of exhibitions such as public exhibitions or trade exhibitions. Furthermore, if a comparative study on the psychological changes of visitors according to the stage of virus spread is conducted, more meaningful results will be drawn. Second, studies using various additional variables related to perceived risk need to be expanded. Especially, it is important to consider how to enhance the core values of on-site exhibitions through follow-up research on how visitors perceive the core values of exhibitions in the midst of the COVID-19 pandemic. Third, since coexistence with COVID-19 is only two years old, and academic and empirical studies on the quality of quarantine service at exhibitions related to COVID-19 are lacking, more efforts are needed to generalize the measuring scale. It is thought that a systematic approach and consideration are necessary for the setting of an exhibition quarantine system, and further for responding to new viruses in the future.

## Figures and Tables

**Table 1:** Perceived risk of exhibition in the era of COVID-19.

Factor	Item
Functional Risk	I thought that the purpose of visiting the exhibition would not be achieved. I thought I would not be satisfied with the contents of the exhibition. I thought the products I wanted to see would not be on display. I thought the exhibition’s scale would be small. I thought I would not get enough relevant information from the exhibition.
Financial Risk	I thought the exhibition entrance fee would be expensive. I thought the related expenses for visiting to the exhibition would be high. I thought it would cost extra to visit the exhibition. I thought it would take a long time to visit the exhibition. I thought it would take a lot of time to travel from my house to the exhibition hall. I thought my time was wasted due to unexpected problems.
Physical Risk	I thought I was likely to be exposed to COVID-19 at the exhibition. I thought I would be able to come into contact with COVID-19 infected person at the exhibition. I thought there could be something wrong with my health after visiting the exhibition. I thought that the exhibition organizers would not properly comply with COVID-19 quarantine measures. I thought that other visitors would not follow COVID-19 quarantine rules properly.
Social Risk	I thought that people around me would feel bad about visiting exhibition due to COVID-19. I thought that visiting an exhibition in a COVID-19 situation could harm the people around me. I thought that visiting an exhibition in a COVID-19 situation would make me nervous.

**Table 2:** COVID-19 quarantine service quality of exhibition hall.

Factor	Item
COVID-19 Quarantine Service Quality	Distance signs and partitions have been installed in the exhibition hall. A wide distance was maintained for air circulation. Exhibitor booths were arranged in consideration of distance. Access control and health check-up were implemented. Management guidelines such as restrictions on admission of those judged unsuitable for medical examination were operated. As a multi-use facility, cleaning, sanitation, and disinfection were well managed. Sanitary and hand washing areas were provided. Non-contact policy enforcement and related technologies were in operation. Ventilation and filtration in the exhibition hall were well done. Exhibitors and organizers cleaned and disinfected well. Communication with visitors regarding quarantine went well. Catering and indoor food were provided as take-out food. The flow of visitors was well managed. A registration process was implemented to reduce face-to-face contact. The number of participants in exhibition halls and conference rooms was managed. Safety rules were provided for easy understanding. Temporary isolation space and medical service points were established. Reporting procedures by health authorities, such as checking visitor information, were being managed. News monitoring and response procedures were established. On-site problem solving and questions and answers from visitors were well done. The movement of visitors was monitored in real time and response procedures were followed. Quarantine products were prepared in the exhibition hall. It seems that the guidelines for managing overseas arrivals are working well. A manager in charge of quarantine was designated.

**Table 3:** Switching intention of exhibition visitors.

Factor	Item
Switching Intention	I would like to refuse to visit the exhibition if possible. I am against visiting exhibitions. I am dissatisfied with my visit to the exhibition. I am critical of visiting exhibitions. I think it is more efficient not to visit the exhibition. I prefer to visit online exhibitions rather than on-site exhibitions.

**Table 4:** Exploratory factor analysis and reliability.

Factor	Item	Factor Loading				Cronbach’s α
Physical Risk	PR12	**0.853**	0.071	0.089	0.208	0.906
	PR13	**0.804**	0.073	0.083	0.313	
	PR15	**0.794**	0.086	0.201	0.116	
	PR14	**0.767**	0.158	0.168	0.298	
	PR16	**0.726**	0.213	0.155	0.271	
Functional Risk	PR2	0.075	**0.878**	0.148	0.139	0.884
	PR3	−0.031	**0.841**	0.095	0.032	
	PR1	0.08	**0.834**	0.113	0.224	
	PR4	0.132	**0.763**	−0.042	−0.029	
	PR5	0.262	**0.746**	0.111	−0.121	
Financial Risk	PR7	0.005	0.097	**0.858**	0.244	0.791
	PR8	0.086	0.11	**0.85**	0.108	
	PR9	0.251	−0.016	**0.692**	−0.087	
	PR6	0.339	0.29	**0.573**	0.148	
Social Risk	PR17	0.417	0.093	0.158	**0.775**	0.919
	PR19	0.497	0.048	0.144	**0.755**	
	PR18	0.536	0.051	0.112	**0.754**	
Eigenvalue		4.112	3.518	2.489	2.244	
Variance (%)		24.187	20.695	14.64	13.197	
Cumulative Variance (%) = 72.718						
Kaiser–Meyer–Olkin Measure of Sampling Adequacy = 0.855						
Bartlett‘s Test of Sphericity: χ^2^ = 1893.322, df = 136, *p* < 0.01						

PR = perceived risk; factor loadings of 0.5 or higher are in bold.

**Table 5:** Correlation coefficients between variables.

Variable	1	2	3	4	5	6
Functional Risk (1)						
Financial Risk (2)	0.282 **					
Physical Risk (3)	0.285 **	0.404 **				
Social Risk (4)	0.204 **	0.376 **	0.326 **			
Service Quality (5)	0.044	0.012	−0.083	0.273 **		
Switching Intention (6)	0.285 **	0.318 **	0.241 **	0.227 **	−0.315 **	
M	2.711	2.874	2.889	2.936	3.596	2.145
SD	0.754	0.736	0.817	0.895	0.475	0.743

** *p* < 0.01.

**Table 6:** Results of multiple regression using perceived risk to predict COVID-19 quarantine service quality.

Model	*B*	*SE*	ß	*t*	*p*	VIF	*R* ^2^	*F*
Functional Risk	0.045	0.052	0.071	0.861	0.391	1.131	0.082	3.587 **
Financial Risk	0.011	0.057	0.017	0.192	0.848	1.263		
Physical Risk	−0.102	0.068	−0.175	−1.488	0.139	2.270		
Social Risk	0.353	0.142	0.244	2.484	0.014 *	2.154		

* *p* < 0.05, ** *p* < 0.01.

**Table 7:** Results of multiple regression using perceived risk to predict switching intention.

Model	*B*	*SE*	ß	*t*	*p*	VIF	*R* ^2^	*F*
Functional Risk	0.193	0.076	0.196	2.553	0.012 *	1.131	0.152	7.275 **
Financial Risk	0.219	0.082	0.217	2.667	0.008 **	1.263		
Physical Risk	0.040	0.099	0.044	0.404	0.687	2.270		
Social Risk	0.061	0.088	0.074	0.695	0.488	2.154		

* *p* < 0.05, ** *p* < 0.01.

**Table 8:** Results of multiple regression using COVID-19 quarantine service quality to predict switching intention.

Model	*B*	*SE*	ß	*t*	*p*	*R^2^*	*F*
COVID-19 Quarantine Service Quality	−0.492	0.116	−0.315	−4.258	0.000 ***	0.099	18.133 ***

*** *p* < 0.001.

## Data Availability

The data presented in this study are available on request from the corresponding author.

## References

[1] Lee T.S., Kim K.Y. (2020). The influence of tourism and MICE industry on the COVID-19 and future countermeasures: Focusing on tourism and MICE industry in Busan. J. Tour. Leis. Res..

[2] Park H.Y., Hwang S.M. (2020). Study on the MICE selection attributes for face-to-face events in Post COVID-19 era using modified IPA: Focusing on a quarantine and safety management factor. Trade Exhibit. Res..

[3] Dong A.I. Postponed by 75% of the Exhibition after Corona: About 320 Billion Won in Damage in the First Half. https://bizn.donga.com/STUDIO/home/article/all/20200618/101574416/1.18.

[4] Ha H.K., Lee H.C. (2020). Analysis on the determinants of demand for exhibition visitors focused on exhibition involvement in the era of COVID-19. J. Tour. Manag. Res..

[5] UFI The Global Recovery Insights 2021 Report. https://www.ufi.org/mediarelease/the-global-recovery-insights-2021-report-published-the-road-to-recovery/.

[6] Choi J.W., Hwang Y.J., Lee H. (2021). The effect of risk perception of COVID-19 on domestic travel intention: Focusing on protection motivation theory. J. Tour. Stud..

[7] Reisinger Y., Mavondo F. (2005). Travel anxiety and intentions to travel internationally: Implications of travel risk perception. J. Travel Res..

[8] Kim Y.N., Hong S.H. (2020). An exploratory study on optimistic bias in risk perception of COVID 19: Perspectives of Jeju visitors. J. Tour. Sci..

[9] Rho S.H., Yi C.G. (2021). The effect of tourists’ risk perception due to the COVID-19 on choice intention of accommodations in tourist sites. J. Hosp. Stud..

[10] Wu E.H., Law R., Jiang B. (2010). The impact of infectious diseases on hotel occupancy rate based on independent component analysis. Int. J. Hosp. Manag..

[11] Habib S., Hamadneh N.N. (2021). Impact of perceived risk on consumers technology acceptance in online grocery adoption amid covid-19 pandemic. Sustainability.

[12] Hakim M.P., Zanetta L.D.A., da Cunha D.T. (2021). Should I stay, or should I go? Consumers’ perceived risk and intention to visit restaurants during the COVID-19 pandemic in Brazil. Food Res. Int..

[13] Jittrapirom P., Tanaksaranond G. An Exploratory Survey on the Perceived Risk of COVID-19 and Travelling. https://osf.io/preprints/socarxiv/v3g5d/.

[14] Martinelli E., De Canio F., Nardin G. (2021). Consumers’ channel switching behaviour from off-line to on-line: The role of the fear of Covid-19. National Brand and Private Label Marketing Conference.

[15] Matiza T. (2020). Post-COVID-19 crisis travel behaviour: Towards mitigating the effects of perceived risk. J. Tour. Futures.

[16] Ozbilen B., Slagle K.M., Akar G. (2021). Perceived risk of infection while traveling during the COVID-19 pandemic: Insights from Columbus. OH. Transp. Res. Interdiscip. Perspect..

[17] Palau-Saumell R., Matute J., Derqui B., Meyer J.H. (2021). The impact of the perceived risk of COVID-19 on consumers’ attitude and behavior toward locally produced food. Br. Food J..

[18] Pham V.K., Do Thi T.H., Ha Le T.H. (2020). A study on the COVID-19 awareness affecting the consumer perceived benefits of online shopping in Vietnam. Cogent Bus. Manag..

[19] Sánchez-Cañizares S.M., Cabeza-Ramírez L.J., Muñoz-Fernández G., Fuentes-García F.J. (2021). Impact of the perceived risk from Covid-19 on intention to travel. Curr. Issues Tour..

[20] Haddock C. (1993). Managing Risks in Outdoor Activities.

[21] Bauer R.A. (1960). Consumer Behavior as Risk Taking. J. Service Sci. Manage..

[22] Oh M.Y., Oh M.S. (2013). An exploratory study on optimistic bias in risk perception of tourist destination. Int. J. Tour. Hosp. Res..

[23] Park H.J., Lee C. (2020). Effects of cultural differences on the relationship between perceived risk and brand loyalty: Focusing on the moderating effects of collectivism and risk avoidance. Int. Bus. J..

[24] Sönmez S.F., Graefe A.R. (1998). Influence of terrorism risk on foreign tourism decisions. Ann. Tour. Res..

[25] Mäser B., Weiermair K. (1998). Travel decision-making: From the vantage point of perceived risk and information preferences. J. Travel Tour. Mark..

[26] Choi H.S., Kim J.H., Lee S.G. (2010). Relationship between perceived risk and tourism information search of outbound tourists. Int. J. Tour. Manag. Sci..

[27] Lee K.J., Han Y.K. (2007). A study on the effect of perceived risk and quality on a traveler′s behavior. Int. J. Tour. Hosp. Res..

[28] Oh J.M., Yoon Y.H., Yoon Y.S., Lee H.R. (2014). A study on influence of perceived risk of MICE destination on visiting switching behavior and loyalty. Korea Sci. Art Forum.

[29] Ryu I.P., Kim Y.J. (2011). A study on the effects of tourists’ perceived risk on their behavioral attitudes and purchase intentions. Int. J. Tour. Manag. Sci..

[30] Korea Tourism Organization (2020). AIPC, ICCA, UFI-Requirements for Resumption of COVID-19 Related Business Events.

[31] UFI Good Practice Guide: Addressing COVID-19 Requirements for Re-Opening Business Events. https://www.ufi.org/archive-research/good-practice-guide-addressing-covid-19-requirements-for-re-opening-business-events/.

[32] Kim H.J. (2016). A study on the relationship among service quality, experiential value, satisfaction and revisit intention. Trade Exhibit. Res..

[33] Zeithaml V.A., Berry L.L., Parasuraman A. (1996). The behavioral consequences of service quality. J. Mark..

[34] Jang M.H., Oh I.G. (2013). The influence relationship with the participation purpose, attributes of trade fair choice and a satisfaction of exhibitors. Trade Exhibit. Res..

[35] Kim D.H., Lee L.N. (2021). A study on influence of the service quality of exhibition and perceived crowding on satisfaction-Focused on visitors of Korea Travel Expo 2019. Trade Assoc. Res..

[36] Wong J.Y., Li T.H., Chen A., Peng N. (2017). The effects of trade show environments on visitors. Event Manag..

[37] Wu H.C., Cheng C.C., Ai C.H. (2016). A study of exhibition service quality, perceived value, emotion, satisfaction, and behavioral intentions. Event Manag..

[38] Wu H.C., Li T. (2015). An empirical study of the effects of service quality, visitor satisfaction, and emotions on behavioral intentions of visitors to the museums of Macau. J. Qual. Assur. Hosp. Tour..

[39] Kim D.I., Lee S.K., Jin H.S. (2012). An analysis of structural relationship among the effect traveler’s satisfaction by preference attributes of overseas travel products on the attitude, loyalty and switching intention to travel agency: Focusing on the difference in travel agency scale. Int. J. Tour. Hosp. Res..

[40] Jones M.A., Mothersbaugh D.L., Beatty S.E. (2002). Why customers stay: Measuring the underlying dimensions of services switching costs and managing their differential strategic outcomes. J. Bus. Res..

[41] Lee Y.A. (2021). The effect of psychological contract violation on switching intention of hotel guests: Focused on the moderating role of hotel credibility. Food Serv. Ind. J..

[42] Bitner M.J., Booms B.H., Tetreault M.S. (1990). The service encounter: Diagnosing favorable and unfavorable incidents. J. Mark..

[43] Yeo H.K. (2007). The effect of subjective norms, self efficacy, switching attitudes, intention, behavior and revisit intention in marine tourist. J. Tour. Leis. Res..

[44] Bansal H.S., Taylor S.F. (1999). The service provider switching model (APSM): A model of consumer switching behavior in the services industry. J. Serv. Res..

[45] Lee D.H., Yun E.J. (2021). The effect of exhibition service quality of medical tourism in attendance satisfaction and behavioral intention. Event Manag..

[46] Liang L.J., Choi H.C., Joppe M. (2018). Exploring the relationship between satisfaction, trust and switching intention, repurchase intention in the context of Airbnb. Int. J. Hosp. Manag..

[47] Keaveney S.M. (1995). Customer switching behavior in service industries: An exploratory study. J. Mark..

[48] Kwon H.J., Ji Y.H. (2020). A study on the decision factors of the hotel customers′ switching tntention by COVID-19. J. Hotel Resort.

[49] Nunnally J.C., Bernstein I.H. (1994). Psychometric Theory.

[50] Menard S.W. (2002). Applied Logistic Regression Analysis.

